# Usnic Acid Treatment Changes the Composition of Mycobacterium tuberculosis Cell Envelope and Alters Bacterial Redox Status

**DOI:** 10.1128/mSystems.00097-21

**Published:** 2021-05-04

**Authors:** Elwira Sieniawska, Rafal Sawicki, Wieslaw Truszkiewicz, Andrey S. Marchev, Milen I. Georgiev

**Affiliations:** a Medical University of Lublin, Chair and Department of Pharmacognosy, Lublin, Poland; b Medical University of Lublin, Chair and Department of Biochemistry and Biotechnology, Lublin, Poland; c Bulgarian Academy of Sciences, The Stephan Angeloff Institute of Microbiology, Laboratory of Metabolomics, Plovdiv, Bulgaria; d Center of Plant Systems Biology and Biotechnology, Plovdiv, Bulgaria; Chan Zuckerberg Biohub

**Keywords:** lipids, metabolomics, LC-MS, NMR, natural products, sigma factors, stress response

## Abstract

Mycobacterium tuberculosis developed efficient adaptation mechanisms in response to different environmental conditions. This resulted in the ability to survive in human macrophages and in resistance to numerous antibiotics. To get insight into bacterial responses to potent antimycobacterial natural compounds, we tested how usnic acid, a lichen-derived secondary metabolite, would influence mycobacteria at transcriptomic and metabolomic levels. The analysis of expression of sigma factors revealed a profound impact of usnic acid on one of the primary genetic regulatory systems of M. tuberculosis. Combined liquid chromatography-mass spectrometry and nuclear magnetic resonance analyses allowed us to observe the perturbations in metabolic pathways, as well as in lipid composition, which took place within 24 h of exposure. Early bacterial response was related to redox homeostasis, lipid synthesis, and nucleic acid repair. Usnic acid treatment provoked disturbances of redox state in mycobacterial cells and increased production of structural elements of the cell wall and cell membrane. In addition, to increase the number of molecules related to restoration of redox balance, the rearrangements of the cell envelope were the first defense mechanisms observed under usnic acid treatment.

**IMPORTANCE** The evaluation of mechanisms of mycobacterial response to natural products has been barely studied. However, it might be helpful to reveal bacterial adaptation strategies, which are eventually crucial for the discovery of new drug targets and, hence, understanding the resistance mechanisms. This study showed that the first-line mycobacterial defense against usnic acid, a potent antimicrobial agent, is the remodeling of the cell envelope and restoring redox homeostasis. Transcriptomic data correlated with metabolomics analysis. The observed metabolic changes appeared similar to those exerted by antibiotics.

## INTRODUCTION

Mycobacterium tuberculosis is a persistent causative agent of tuberculosis. Its characteristic cell envelope composition and metabolic ability to adapt to the extreme conditions found in macrophages make eradication of these bacteria challenging. M. tuberculosis developed numerous survival strategies, such as the ability to utilize several carbon sources (1), ability to change cell wall hydrophobicity during invasion (2), or ability to respond to various stresses through complicated, regulatory networks of transcription factors (3). The observations of bacterial responses at genetic and metabolic levels are crucial for the identification of new molecular targets that can be hit by active molecules. What is more, the reprogramming of cell metabolism was recognized as an effective bacterial resistance strategy, manifested by a number of resistance pathways (4). These resistance/adaptation pathways can be successfully monitored by application of metabolomics. Metabolomics depicts the products of transcriptional, translational, and enzymatic activity of the cell and, thus, provides insight into metabolic states of bacteria under physiological and stress conditions (5). The metabolomic approach was already successfully applied to describe metabolic signatures of *Mycobacterium* associated with different growth stages (6), stresses (7, 8), resistance (9), and modes of action of antibiotics (10–12).

The scaffolds of antibiotics are often of natural origin; therefore, natural products are still of high interest in combating tuberculosis (13). Many antibiotics, including molecules active against M. tuberculosis, were isolated from soil bacteria or fungi (14). For example, usnic acid exhibited very good antimycobacterial activity (MICs) against *M. aurum* (32 μg/ml) (15), Mycobacterium avium (16 μg/ml) (16), Mycobacterium chelonae (25 μg/ml) (17), Mycobacterium fortuitum (50 μg/ml) (17), Mycobacterium kansasii (12.5 μg/ml) (17), and M. tuberculosis H37Rv (from 1.56 to 62.5 μg/ml) (16–19). Usnic acid is a dibenzofurandione found only in lichens as a crystalline yellow cortical pigment and is poorly soluble in water (15). Although a few studies demonstrated that usnic acid can increase bacterial membrane permeability (20), inhibit nucleic acids synthesis (21), inhibit efflux pumps, and alter fatty acid and peptidoglycan production (22) in some bacteria, its antibacterial mechanism of action is not fully elucidated. Moreover, the antimycobacterial activity of this compound was not explained so far. To fill this gap, and to reveal M. tuberculosis responses to usnic acid treatment, we applied untargeted metabolomics supported by the evaluation of expression levels of three members of the sigma (σ) family.

Through the expression of accessory sigma factors, bacteria are ready to respond to different environmental stimuli (23). Changes in RNA polymerase-sigma factor (holoenzyme) interaction lead to the initiation of transcription of particular gene sets and, consequently, changes the metabolic pathways (3). The sigma family is composed of 13 factors, one essential under normal growth conditions, housekeeping SigA, one stress-responsive SigB, and 11 accessory factors, which are clustered in functional groups. The complex, three-level topology of this network includes master regulators and coregulators. In addition, sigma factors are posttranslationally inactivated by their cognate anti-sigma factors (3, 23). Such a regulatory mechanism enables dynamic and multilayer signal processing, resulting in the effective reprogramming of cell metabolism. Therefore, the evaluation of expression levels of sigma factors provides a global view on the bacterial response to compounds inhibiting their growth. In this work, we integrated *sigA*, *sigB*, and *sigG* expression with metabolomics aiming to reveal early bacterial responses to usnic acid treatment.

## RESULTS

### LC-MS metabolite characterization.

LC-MS–XCMS workflow (liquid chromatography-mass spectrometry followed by data processing in XCMS Online) indicated metabolic pathways that were altered under usnic acid treatment (Table 1). Six pathways were related to nucleotide metabolism, with cytidine- and uridine-based molecules that were upregulated, while adenine- and guanine-based ones were downregulated. Upregulation was also noticed for biosynthesis of riboflavin, adenosylcobalamin, arginine, factor 420, and mycolate. Considering the functions of dysregulated metabolites, the listed pathways can be divided into those related to redox reactions and pathways related to lipid synthesis and nucleic acid repair and replication.

**TABLE 1:** Activity network/connections obtained for bacteria under usnic acid treatment

KEGG pathway and metabolite	Total	No. of hits, direction of fold change	*P* value
Pyrimidine deoxyribonucleotide *de novo* biosynthesis I and III	4	4	0.0002
CDP		45.6, up	
CTP		2.3, up	
dCDP		385.4, up	
dUTP		2.1, up	
CMP phosphorylation	3	2	0.00562
CDP		45.6, up	
CTP		2.3, up	
Pyrimidine deoxyribonucleotide dephosphorylation	2	2	0.00222
5-Hydroxy-CTP		1.7, up	
dUTP		2.1, up	
Purine deoxyribonucleoside degradation I	4	2	0.01172
2′-Deoxyguanosine		2.7, down	
Adenine		1.6, down	
*S*-Methyl-5′-thioadenosine degradation II and IV	2	2	0.00222
Adenine		1.6, down	
*S*-Methyl-5′-thioadenosine		1.6, down	
Adenine and adenosine salvage III	4	2	0.01566
Adenine		1.6, down	
Inosine		2.3, down	
Flavin biosynthesis I (bacteria and plants)	2	2	0.00617
FMN		125.8, up	
Riboflavin		37.0, up	
Factor 420 biosynthesis	4	2	0.01172
7,8-Didemethyl-8-hydroxy-5-deazariboflavin		68.2, up	
5,10-Methylene-tetrahydromethanopterin		308.6, up	
Arginine biosynthesis II (acetyl cycle)	4	2	0.01172
AMP		1.4, up	
* N*-Acetyl-l-ornithine		9.3, up	
Adenosylcobalamin biosynthesis from cobyrinate *a*,*c*-diamide II	5	2	0.02109
FMN		125.8, up	
Nicotinate		1.6, up	
Cob(II)yrinate *a*,*c*-diamide biosynthesis II (late cobalt incorporation)	5	2	0.02296
Precorrin-8x		7.2, up	
* S*-Adenosyl-l-homocysteine		12.9, up	
Mycolate biosynthesis	6	2	0.03215
CoA		12.1, up	
* S*-Adenosyl-l-homocysteine		12.9, up	

**FIG 1:**
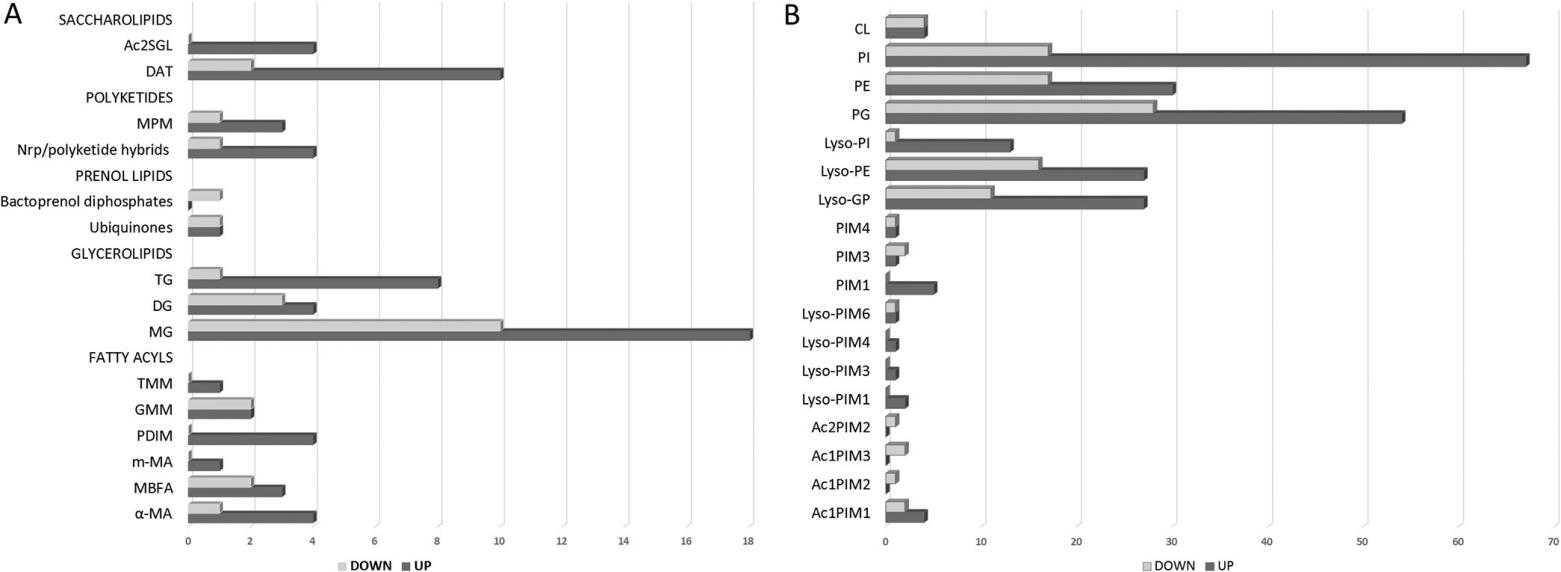
Number of dysregulated lipids with breakdown for classes and subclasses. (A) Fatty acyls, glycerolipids, prenol lipids, polyketides, and saccharolipid class. (B) Glycerophospholipids. α-MA, mycolic acids; MBFA, methyl branched fatty acids; m-MA, metoxy mycolic acids; PDIM, phthiocerol dimycocerosates; GMM, glucose monomycolates; TMM, trehalose monomycolates; MG, monoacylglycerols; DG, diacylglycerols; TG, triacylglycerols; MPM, mannosyl-b1-phosphomycoketides; DAT, diacyltrehaloses; Ac2SGL, diacylated sulfolipid; PI, diacylglycerophosphoinositols; AcPIMs, acylated diacylglycerophosphoinositolmannosides; Lyso-GP, monoacylglycerophosphoglycerols; PIMs, diacylglycerophosphoinositolmannosides; Lyso-PIMs, monoacylglycerophosphoinositolmannosides; PG, diacylglycerophosphoglycerols; Lyso-PE, monoacylglycerolphosphoethanolamines; Lyso-PI, monoacylglycerophosphoinositols; PE, diacylglycerolphosphoethanolamines; CL, diacylglycerophosphoglycerophosphodiradylglycerols.

The LC-MS–XCMS workflow was further supported by lipid analysis in the MS-based lipid(ome) analyzer and molecular platform (MS-LAMP). The annotated metabolites were assigned to six classes of lipids: fatty acyls, glycerolipids, glycerophospholipids, polyketides, prenol lipids, and saccharolipids. The correlation of the outputs from XCMS and MS-LAMP revealed lipids dysregulated under usnic acid treatment. The number of up/downregulated compounds is shown in Fig. 1, while the fold change of individual compounds in classes/subclasses is included in the supplemental material.

In the class of fatty acyls, predominant upregulation was noticed. It was the most pronounced for α-mycolic acids α-MA (C76), α-MA (C77), and α-MA (C85), which increased 219-, 86-, and 5-fold, respectively, and for methyl branched fatty acids (MBFA) phthioceranic acid (C36), mycocerosic acid (C32), and mycolipanolic acid (C24), which increased 7-, 31-, and 44-fold, respectively. Phthiocerol and phthiodiolone dimycocerosates (PDIM) and trehalose monomycolates (TMM) were also overexpressed.

More than half of the detected glycerolipids were only slightly dysregulated (up to 2-fold). Monoacylglycerols (MG), diacylglycerols (DG), and triacylglycerols (TG) were up- and downregulated, and most of the compounds showed overexpression, which was very significant for several MG [MG (16:1); MG (20:0); MG (22:1); MG (21:0); increased 14-, 51-, 145-, and 378-fold, respectively] and two TG [TG (52:1) and TG (48:3); increased 12- and 85-fold, respectively]. The highest fold change down (maximum, 11-fold) was observed for DG.

Glycerophospholipids were the most abundant class of detected lipids, containing 342 compounds. The observed dysregulation caused by usnic acid treatment was up to 2-fold for 225 compounds. Seventy-five compounds were influenced between 2 and 5-fold, while remaining compounds were significantly upregulated more than two hundred times. The most abundant subclasses were diacylglycerophosphoinositols (PI), diacylglycerophosphoglycerols (PG), diacylglycerolphosphoethanolamines (PE), and their corresponding monoacylglycero derivatives, with prevalent numbers of overexpressed molecules. The usnic acid treatment caused a significant change in the levels of PG, highly increasing the levels of some compounds [PG (R1CO2H + R2CO2H = 46:0), 490-fold up; PG (R1CO2H + R2CO2H = 35:1), 20-fold up; PG (R1CO2H + R2CO2H = 45:0), 5-fold up] while decreasing the levels of others [PG (R1CO2H + R2CO2H = 33:0), 254-fold down; PG (R1CO2H + R2CO2H = 32:0), 36-fold down; PG (R1CO2H + R2CO2H = 30:1), 25-fold down; PG (R1CO2H + R2CO2H = 31:0), 15-fold down). Lyso-GP also was affected, with upregulation of up to 20-fold and downregulation of up to 500-fold for Lyso-PG (RCO2H = 16:1). CL (2R1CO2H  + 2R2CO2H = 63:1) was overexpressed 60-fold up. For the majority of detected PE, the dysregulation was only slight, but PE (R1CO2H + R2CO2H = 31:1) and PE (R1CO2H + R2CO2H = 37:0) decreased by 13- and 6-fold, respectively. Lyso-PE were overexpressed up to 215-fold (Lyso-PE [R1CO2H = 18:0]) and downregulated up to 10-fold (Lyso-PE [R1CO2H = 16:1]). The usnic acid treatment resulted in noticeable dysregulation in the family of inositol-based compounds (PI, Lyso-PI, diacylglycerophosphoinositolmannosides [PIM], Lyso‐PIM, and acylated diacylglycerophosphoinositolmannosides [AcPIM]). The most pronounced changes were observed for the PI subclass, among which 8 compounds were influenced from 7- to 42-fold, with the highest upregulation (42-fold) noticed for PI (R1CO2H + R2CO2H = 36:3). Only two PI, PI (R1CO2H + R2CO2H = 35:2) and PI (R1CO2H + R2CO2H = 31:0), and one Lyso-PI [Lyso-PI (RCO2H = 16:0)], were very significantly downregulated by 19-, 12-, and 12-fold, respectively. In the Lyso-PIM subclass, Lyso-PIM4 (RCO2H = 18:2) was upregulated 45-fold, Lyso-PIM3 (RCO2H = 17:0) 23-fold, and Lyso-PIM6 (RCO2H = 16:0) 6-fold.

Polyketides, prenol lipids, and saccharolipids were also affected. The significant downregulation was noticed for mannosyl-b1-phosphomycoketide (MPM) (C34) (254-fold), while mycobactin Mbt-Fe (*R* = 18:0) and menaquinone MK S881 were upregulated up to 4 and 4.5 times, respectively. Among all saccharolipids influenced by usnic acid treatment, only two compounds were downregulated. Upregulation was observed for 2,3-di-O-acyltrehaloses DAT1 (up to 5-fold for DAT1 [C56]) and DAT2 (up to 13-fold for DAT2 [C58]) and for diacylated sulfolipids (up to 3-fold for Ac2SGL [C62]).

### NMR metabolite characterization.

**FIG 2:**
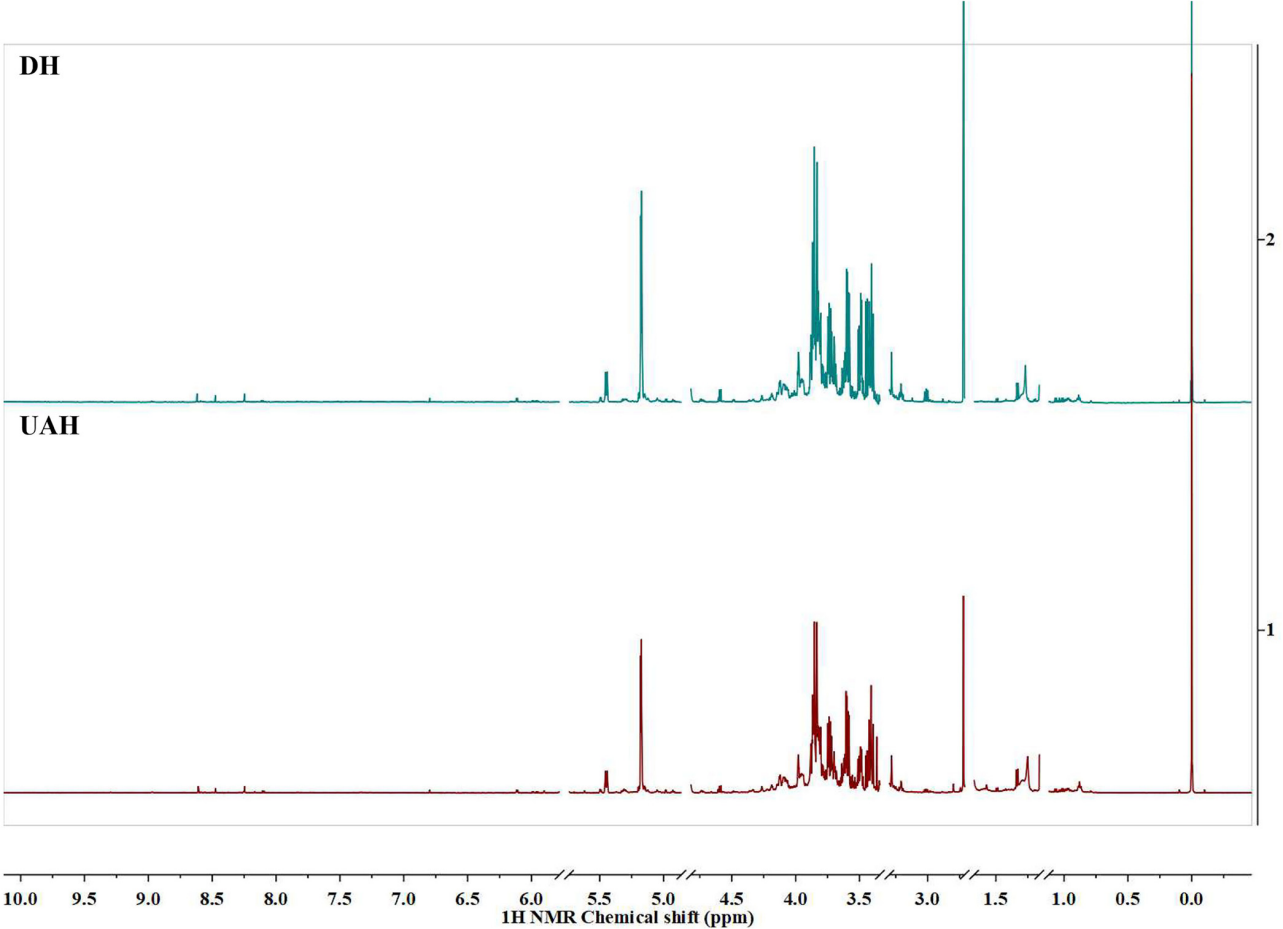
^1^H NMR (600 MHz) spectra of M. tuberculosis H37Ra control samples (DH) and samples treated with usnic acid (UAH). The residual signals of solvents and usnic acid were removed from the spectrum.

The metabolite alterations in M. tuberculosis H37Ra as a result of the treatment with usnic acid were also distinguished through the application of one-dimensional (1D)- and 2D-nuclear magnetic resonance (NMR) analyses. Qualitative and quantitative differences in the metabolites were found between the control and usnic acid-treated groups (Fig. 2). The most significant differences were found in the aliphatic region (*δ* 0.0 to 3.0 ppm), where abundant signals were assigned to several amino acids, such as alanine, glutamine, leucine, proline, threonine, and valine. Within the amino acid pool, the relative amounts of glutamine, proline, and serine decreased as a result of the usnic acid treatment (Table 2). The review of carbohydrate region (*δ* 3.0 to 5.5 ppm) revealed several metabolite alterations as well, expressed in decreased amounts of glucose and xylose. The amounts of dimethylamine, GABA, and IMP also decreased in the treated sample.

**TABLE 2:** Chemical shifts (*δ*) and coupling constants (*J*) of the metabolites, identified by their relevant ^1^H NMR spectra

Metabolite (reference[s])	Chemical shift (ppm)	Coupling constant (Hz)	DH^*a*^	UAH^*a*^
Amino acid				
Alanine (52, 53)	1.49	(d, *J *= 7.2)	+	+
Glutamine (52, 53)	2.14/2.39	(m)/(m)	++	+
Leucine (54)	0.98/0.99/3.73	(d, *J *= 6.3)/(d, *J *= 6.3)/(m)	+	+
Proline (55)	4.13/2.40/2.12/2.05/3.34/3.41	(dd, *J *= 6.3; 8.7)/(m)/(m)/(m)/(m)/(m)	++	+
Serine (55)	3.98	(m)	++	+
Threonine (52, 53)	1.33	(d, *J *= 6.9)	+	+
Valine (52, 53)	1.01/1.07	(d, *J *= 7.3)/(d, *J *= 7.1)	+	+
Carbohydrate				
α-Glucose (52, 53)	5.18	(d, *J *= 3.8)	++	+
β-Glucose (52, 53)	4.59	(d, *J *= 7.9)	++	+
Galactose (56)	5.15	(d, *J *= 3.6)	+	+
Raffinose (57)	5.49	(d, *J *= 3.7)	+	+
Xylose (58)	3.41/3.63/4.60/5.19	(t, *J *= 9.5)/(m)/(d, *J *= 7.9)/(d, *J *= 4.0)	++	+
Organic acid				
Formic acid (52, 53)	8.47	(s)	++	+
Alcohol				
Glycerol (54)	3.54/3.56/3.58/3.60	(d, *J *= 6.2)/(d, *J *= 6.5)/(d, *J* =3.8)/(d, *J* =3.8)	++	+
Other				
Choline (52, 53)	3.22	(s)	−	+
Dimethylamine (59)	2.72	(s)	+++	+
γ-Amino-butyrate (GABA) (52)	1.90/2.30/3.01	(m)/(t, *J *= 7.3)/(t, *J *= 7.4)	++	+
Lecithin (56)	3.12	(s)	+	−
Inosine monophosphate (60)	8.25/8.61	(s)/(s)	++	+

a DH, control (cells + DMSO); UAH, samples treated with usnic acid. Plus and minus signs indicate the presence and absence of metabolite; + to +++ indicate semiquantitative information via relative signal intensity comparison.

**FIG 3:**
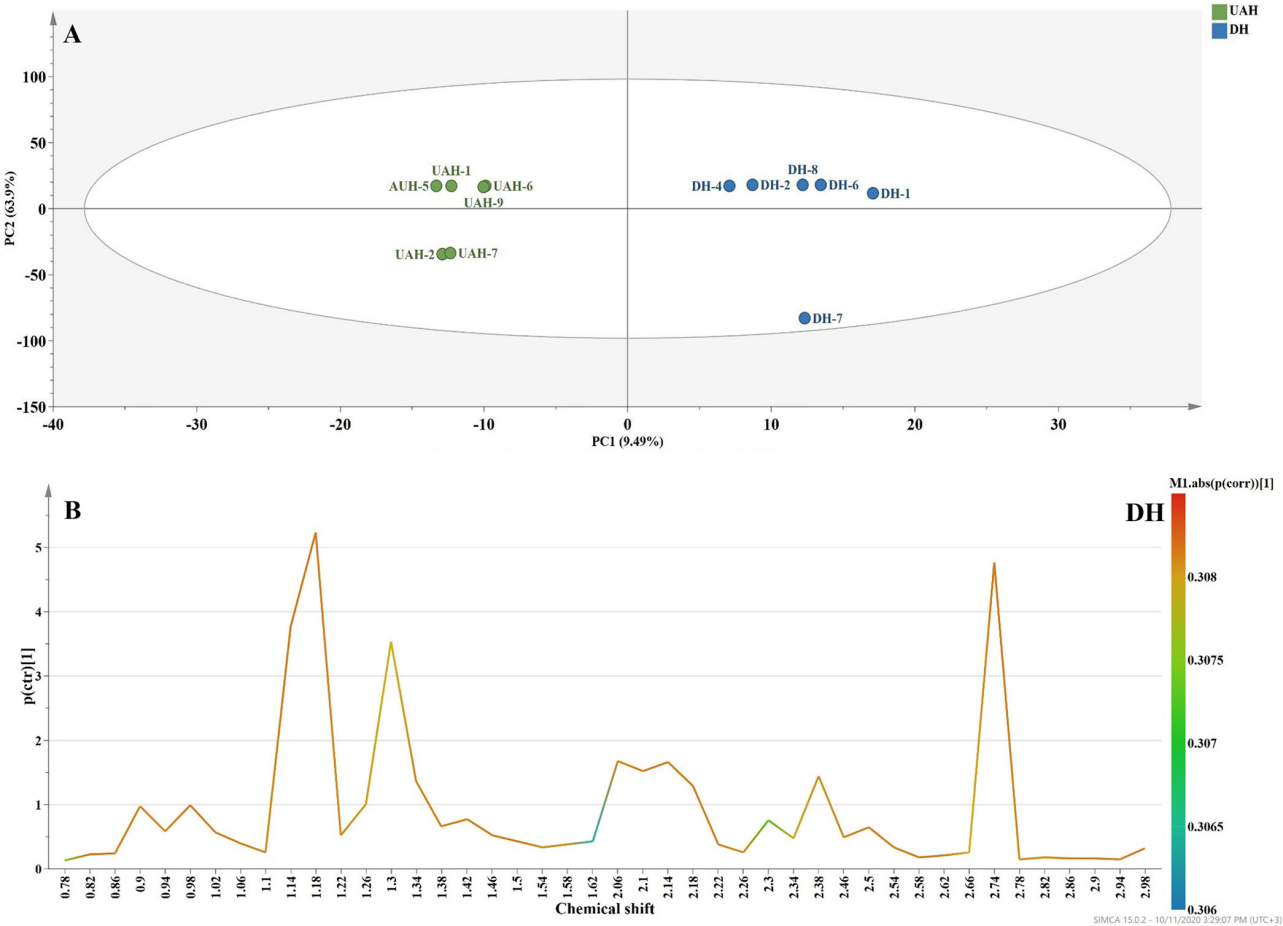
(A) Orthogonal projections to latent structures discriminant analysis (OPLS-DA) statistical model of M. tuberculosis H37Ra control- and usnic acid-treated samples based on the spectral region *δ* 0.0 to 3.0 ppm. (B) Representative S-line based on the OPLS-DA model between the control and usnic acid-treated samples. All signals have positive values, revealing that the molecules in this region are present in larger amounts in the control samples.

Further, the ^1^H NMR data were subjected to orthogonal projections to latent structure discriminant analysis (OPLS-DA) with a focus on the aliphatic region, where most of the differences between the metabolites were observed (Fig. 3A). The applied model clearly clustered the samples into two groups according to principal component 1 (PC1), explaining 70.4% of the variance in the data. The corresponding S-line (Fig. 3B) model between the control and usnic acid-treated strains also confirmed that the identified metabolites (mostly amino acids) were found in larger amounts in the untreated strain.

### Sigma factor expression.

**FIG 4:**
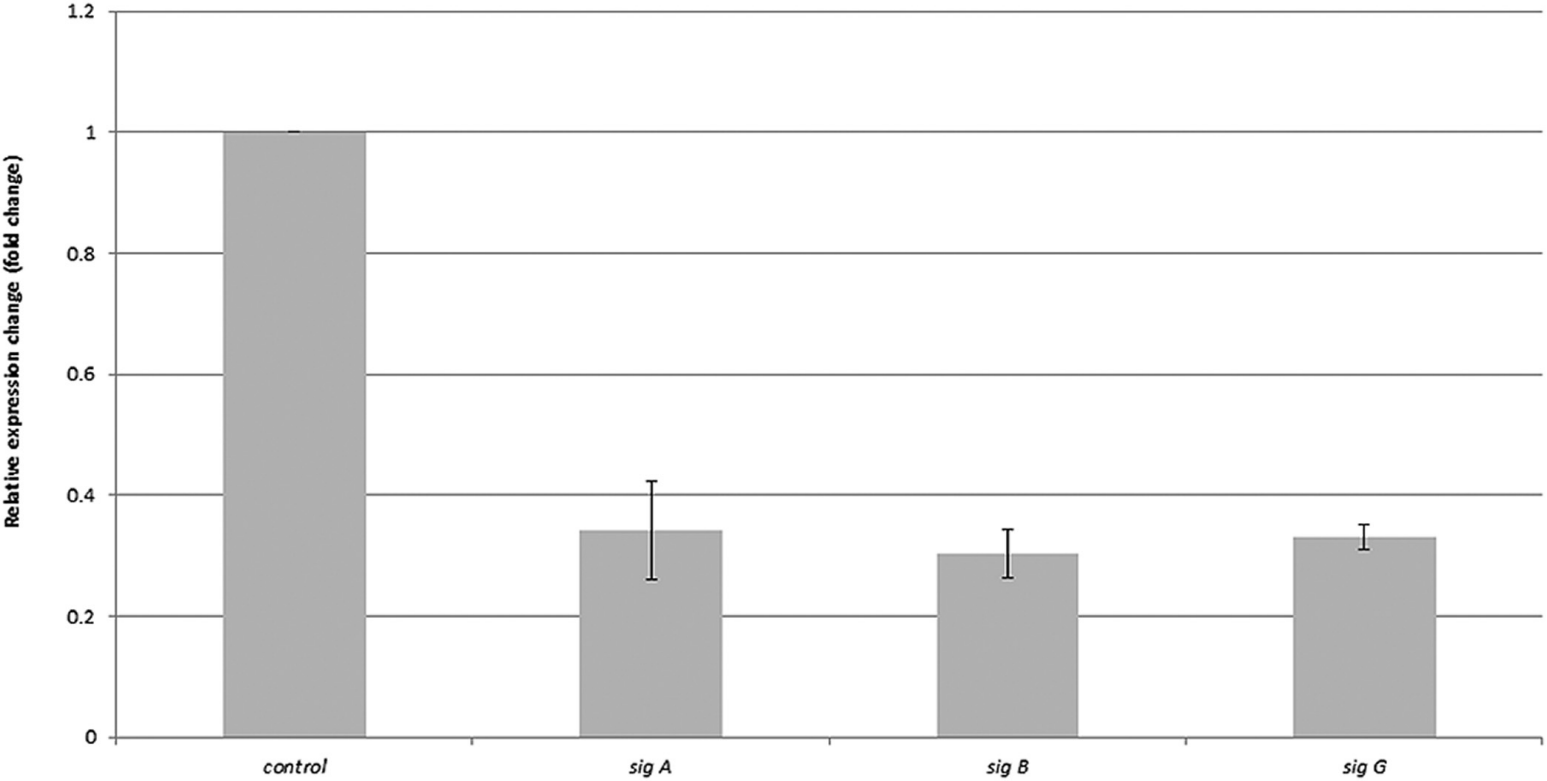
Relative expression change of *sigA*, *sigB*, and *sigB* genes after 24 h of exposure to an effective dose (512 μl/ml) of usnic acid, normalized to 16SmRNA (control). Standard deviations are included as a thin bar.

Two top-level *sigA* and *sigB* genes and one middle-level *sigG* gene form a community involved in regulating such processes as lipid metabolism, hypoxic, and redox response. To shed light on the regulator's expression changes under usnic acid stress, we measured the shifts in *sigA*, *sigB*, and *sigG* transcripts (Fig. 4). All tested sigma factor genes were downregulated over 60% compared to the control. This finding showed the profound impact of usnic acid on one of the primary genetic regulatory systems of M. tuberculosis and is reflected in metabolomic shifts.

## DISCUSSION

Mycobacterium tuberculosis has a high ability to adapt to factors, inhibiting its growth (24). The bacterial response to antibiotics and other substances is manifested by the changes in metabolic pathways and often by substantial remodeling of plasma membrane and outer envelope composition (9, 10, 25, 26). Mycobacteria can also temporarily and reversibly rearrange their basal metabolisms to obtain phenotypic changes protecting them from stress factors (27). This study revealed the alterations in bacterial cells induced by usnic acid treatment. Combined LC-MS and NMR analysis allowed us to observe the perturbations in metabolic pathways, as well as in lipid composition, which took place within 24 h of exposure. Early bacterial response was related to redox homeostasis, lipid synthesis, and nucleic acid repair.

Transcriptomic analysis also indicated the changes in the regulatory network under usnic acid treatment. Chauhan et al. proved that 13 sigma factors create network hierarchy and five communities, coordinately responding to the same stimuli. The network consists of three hierarchical levels. Master regulators SigA, SigB, SigH, and SigM act downstream through the middle (SigE, SigG, SigL, SigJ, and SigF) and lower (SigD, SigK, SigI, and SigC) levels. Sigma factors, together with anti-sigma factors, anti-anti-sigma factors, and transcription factors, create a complex system regulating mycobacterial gene expression. SigA, SigB, and SigC form community-regulating lipid metabolism, hypoxic, and redox responses (3). Observed downregulation of the sigma factor community may explain the number of dysregulated lipids. SigA cooperates with the Rv2034 transcription factor, responsible for regulating lipid metabolism-related genes such as *phoP*, *fadE5*, or *desA2*. The same factor interacts with transcriptional repressor KstR, controlling the expression of genes used for utilizing diverse lipids as energy sources. SigA, SigG, and SigB work in tandem with *lsr2*, coding a DNA-binding protein that has architectural and regulatory roles (28) and protects, among other regulations, mycobacteria against reactive oxygen intermediates. SigB interacted with a DosS member of the two-component regulatory system DevR/DevS (DosR/DosS), involved in the onset of the dormancy response (29) and suggested to act as a redox sensor or a dual oxygen/redox sensor (30).

### Redox reactions.

Both riboflavin and its active form, flavin mononucleotide (FMN), were detected in larger amounts in the treated than in the control group. Flavins work with flavin-dependent monooxygenases. They participate in numerous redox reactions, donating one or two electrons, activating O_2_, and hydroxylating a variety of organic substrates (31, 32). Metabolites involved in factor 420 (F_420_) biosynthesis also were significantly upregulated. F_420_, the deazaflavin derivative, is a redox-active compound working with F_420_-dependent glucose-6-phosphate dehydrogenase. The reaction provides a reduced form of the coenzyme (F_420_H_2_) inside cells, which, in turn, is required for xenobiotics metabolism. The increased biosynthesis of F_420_ is usually linked to a higher demand for reduction of compounds. Hence, the larger amount of factor 420 indicates the imbalance in redox status in bacterial cells. This was further supported by detected upregulation of metabolites involved in arginine biosynthesis through the acetyl cycle. Since arginine deprivation was recognized as a cause of induced oxidative damage in M. tuberculosis, this compound might also be needed for bacterial defense against (ROS)-mediated oxidative stress and DNA damage, which is a common secondary result of bactericidal antibiotics action (33). Another observation was a slightly, but statistically significant, lowered expression of purine nucleoside (2′-deoxyguanosine) and nucleobases (adenine and inosine), which can indicate changes in their metabolism. IMP was detected by NMR in smaller amounts as well. Purine and pyrimidine metabolism is related to oxidative stress (34). For example, the dephosphorylation process is used to degrade and prevent the incorporation of nucleotides modified by the action of reactive oxygen species or xenobiotics (35), and this pathway was annotated as upregulated in our study. Polyamines stabilize nucleic acids and membranes for their integrity maintenance. The downregulated *S*-methyl-5′-thioadenosine (MTA) in this study is a by-product of polyamine biosynthesis and a strong inhibitor of this process. Bacteria treated with usnic acid also produced smaller amounts of GABA, as detected by NMR. Because GABA results from putrescine, which, in turn, is a final product of the polyamine degradation pathway (36), decreased amounts of GABA indicate diminished catabolism of polyamines to compensate for their probable lowered synthesis caused by usnic acid.

### Pathways related to lipid synthesis and nucleic acid repair.

The already-mentioned, upregulated FMN is likewise necessary for adenosylcobalamin biosynthesis. Compounds needed in late cobalt incorporation in this pathway were upregulated, corresponding to detected larger amounts of FMN and suggesting increases in demand/production of adenosylcobalamin. Being a coenzyme B_12_, adenosylcobalamin takes part in several metabolic pathways in M. tuberculosis. It is crucial for methionine biosynthesis, fatty acid metabolism, and DNA repair and replication (37). The observed increase in adenosylcobalamin biosynthesis was indirectly correlated with upregulated mycolate biosynthesis. The synthesis of methionine from homocysteine and 5-methyltetrahydrofolate requires the participation of B_12_-dependent methionine synthase (37). The obtained methionine is a very important intermediate in the formation of mycolic acids. It is used in the activated methyl cycle to supply the pool of reactive methyl groups incorporated into meromycolic chains (38). The possible upregulation in mycolate biosynthesis was also confirmed by the detected increased expression of mycolic acids and corresponding derivatives in the test samples. The correlation with lipid production was observed through pyrimidine nucleotide upregulation. Several forms of pyrimidine nucleotides and deoxynucleotides were overexpressed in bacteria under usnic acid treatment (Table 1). These cytosine- and uracil-based compounds (mainly CTP and UTP) are precursors of nucleic acids; however, they also play important functions in biosynthesis of phospholipids and polysaccharides, respectively (36). Hence, in addition to possible fluctuations in pyrimidine deoxyribonucleotide *de novo* biosynthesis and phosphorylation/dephosphorylation processes, the increased amounts of CDP and CTP correlate with detected intensified production of phospholipids in bacterial cells.

### Alterations in lipids composition. (i) Usnic acid treatment caused increased demand of compounds needed in the formation of more complex structures.

Fatty acyls are comprised of mycolic acids (MA), methyl branched fatty acids (MBFA), and their conjugates with glucose, trehalose, and phthiocerol (39, 40). α-MA, m-MA, MBFA, PDIM, and TMM all were overexpressed. Among α-MA, which constitute more than 70% of all MA (41), α-MA (C76), α-MA (C77), and α-MA (C85) increased very significantly. These unconjugated MA are covalently bounded to arabinogalactan (AG), while their conjugates contribute to the noncovalently linked cell wall layer (41). Upregulation of free MA and in a form of TMM, which is required for transport of MA through the plasma membrane (39), suggests increased MA synthesis. However, it is impossible to say if these compounds were stored inside the cells or already transported to arabinogalactan (AG) acceptors. In the case of methyl-branched fatty acids, most of the annotated compounds were also upregulated, with the highest changes observed for phthioceranic (C36), mycocerosic (C32), and mycolipanolic (C24) acids. These MBFA are the constituents of PDIM as well as polyketides and saccharolipids (42). Their increased synthesis eventually may be linked to higher demand for building blocks needed to form these conjugates.

### (ii) Usnic acid treatment launched intracellular energy resources.

Usnic acid treatment caused significant dysregulation in the class of glycerolipids, which are the main apolar M. tuberculosis lipids and its energy source (43). It appears that glycerolipids were shuffled with a visible tendency to increase the contributions of MG, which are produced only during the degradation of TG and DG (43). As a result of hydrolysis of DA and TG, bacteria produced larger amounts of free fatty acids under usnic acid treatment. These free fatty acids enter β-oxidation, which, in turn, can provide energy and acetyl coenzyme A (CoA). Acetyl CoA is later used for both lipid synthesis via FAS I and anapleurosis of the tricarboxylic acid cycle (44). Thus, it can be hypothesized that usnic acid treatment launched TG energy resources to enhance production of fatty acyls and related structures. This assumption was also supported by NMR analysis results, which revealed lower levels of glycerol, glucose, and xylose in usnic acid-treated samples. This might confirm that bacteria did not assimilate these compounds from the medium but most probably used fatty acids liberated from degraded glycerolipids as carbon and energy sources.

### (iii) Usnic acid increased metabolism of lipids performing structural functions in mycobacterial inner membrane.

Glycerophospholipids appeared to be the most abundant class of detected lipids. They are the largest class of mycobacterial amphipathic polar lipids, which build mycobacterial plasma membrane and constitute precursors for the mycobacterial outer envelope (43). The overexpressed molecules PE, PI, PG, and their corresponding monoacylglycero derivatives contribute to the formation of the mycobacterial cell membrane, which is composed of cardiolipin (CL), PE, PI, and glycosylated and acylated PIs (mainly acylated diacylglycerophosphoinositolmannosides [AcPIM]) (43, 45). PG and, consequently, deacylated monoacylglycerophosphoglycerols (Lyso-GP) are essential for the synthesis of CL, which requires two Lyso-GPs units for one CL molecule (46). The usnic acid treatment caused a significant change in the levels of PG, highly increasing the levels of some compounds while decreasing the levels of others. Lyso-GP also were affected, suggesting a strong rearrangement process within compounds based on glycerophosphoglycerol. At the same time, CL was highly overproduced, indicating the incorporation of Lyso-PG into CL and suggesting the need to restore or strengthen the cell membrane. High fluctuations observed in PE and Lyso-PE classes supported the hypothesis that the metabolism of lipids performing structural functions in the mycobacterial inner membrane was increased by usnic acid.

What is more, usnic acid treatment resulted in noticeable dysregulation in the family of inositol-based compounds (PI, Lyso-PI, PIM, Lyso‐PIM, and AcPIM). Lyso-PI is involved in PI turnover; however, PI not only constitutes the cell membrane but also is used to produce AcPIMs. AcPIMs with up to two mannose residues contribute to cell membrane formation (45, 47), while highly acylated and mannosylated forms are building blocks and anchors for linear and mature branched lipomannan (LM) and lipoarabinomannan (LAM) (43). The expression of all detected diacylglycerophosphoinositolmonomannosides (PIM1) was increased from 1.2 to 3.8 times for treated compared to control bacteria, suggesting a slightly increased pool of these compounds within the cytoplasm. PIM1 can be converted into PIM2 and then AcPIM2 or into AcPIM1 and then AcPIM2 (47). Because PIM2 was not detected in this study, we can assume that the acylation of PIM1 occurred as a first step by an alternative pathway and then further mannosylation took place. Indeed, slightly higher levels of Ac1PIM1 (up to 3.6-fold) were noticed, indicating increased conversion of PIM1 into Ac1PIM1 under usnic acid treatment. Detected products of further mannosylation (Ac1PIM2 and Ac1PIM3) and acylation (Ac2PIM2) were slightly downregulated. Ac1PIM3 is an intermediate in LM formation that is flipped out to the periplasmic side of the plasma membrane, where it is available for further mannosylation (43, 47). The AcPIM molecules with a number of mannose residues greater than 3 were not detected in this study because of limited scanning range (up to 1,700 *m/z*). The degradation products of acylated PIMs, like PIM3 and PIM4, were only negligibly dysregulated, while Lyso-PIM3, Lyso-PIM4, and Lyso-PIM6 were strongly upregulated, the latter being a possible result of increased degradation of polar Ac1PIM4-Ac1PIM6 (elements of LM). The observations described above suggest that increased production of structural elements of the cell membrane is needed to overcome the action of usnic acid.

### Conclusions.

The performed work integrating transcriptomic and metabolomic analysis revealed that usnic acid influenced the expression of sigma factors involved in the general bacterial regulatory network and caused changes in the production of metabolites under stress conditions. It provoked disturbances of redox state in mycobacterial cells and increased production of structural elements of the cell wall and cell membrane. In addition to increased levels of molecules related to the restoration of redox balance, the rearrangements of the cell envelope were the first defense mechanisms observed under usnic acid treatment. This study might be helpful to reveal bacterial adaptation strategies, which are crucial for the discovery of new drug targets and understanding the resistance mechanisms.

## MATERIALS AND METHODS

### Tested organism and culture conditions. (i) Inoculum preparation.

M. tuberculosis H37Ra (ATCC 25177) was grown on Löwenstein-Jensen slopes (BioMaxima, Lublin, Poland) for up to 2 weeks. The bacterial mass was transferred to 5 ml of the fresh Middlebrook 7H9 broth supplemented with 10% albumin-dextrose-catalase (ADC) and 0.2% glycerol (MilliporeSigma, St. Louis, MO) and was vortexed with 1-mm glass beads for 3 min. After 30 min of sedimentation at room temperature, the upper 2 ml was transferred to a sterile tube and left for the next 15 min. One milliliter of supernatant was placed in a sterile tube and was adjusted to a 0.5 McFarland standard with ADC-supplemented Middlebrook 7H9 broth (48).

### (ii) MIC and effective dose determination.

MIC was determined as described by Sawicki et al. (49). Usnic acid (98% purity; Merck, Darmstadt, Germany) was tested in a concentration range from 256 to 2 μg/ml. Serial 2-fold dilutions were prepared in dimethyl sulfoxide (DMSO; MilliporeSigma, St. Louis, MO) using 7H9-S medium for dilution. The final DMSO concentration did not exceed 2% (vol/vol). As reference standards, isoniazid, ethambutol, rifampin, streptomycin, and ciprofloxacin (MilliporeSigma, St. Louis, MO) were used. Final 2-fold dilutions of antibiotics starting from 16 to 0.001 μg/ml were prepared in 7H9-S broth. The round-bottom microwell plates were filled with 50 μl of inoculum, and 50 μl of usnic acid dilution was added to each well. The controls of sterility, growth, and 2% DMSO were included. The plates were sealed with adhesive foil to prevent liquid evaporation and incubated for 8 days at 37°C. After that time, 10 μl of resazurin (alamarBlue; Invitrogen, Carlsbad, CA) solution was added to all wells and incubated for 48 h at 37°C. The MIC was defined as the lowest usnic acid concentration preventing the blue-to-pink change. The effective dose of 512 μg/ml usnic acid for the obtained high-density mycobacterial cultures (approximately 10^9^ CFU/ml) was determined in preliminary experiments (data not shown) and inhibited the growth of bacteria by 50%.

### (iii) M. tuberculosis exposure to usnic acid.

The exposure to usnic acid was performed according to general rules of metabolomic experiments (50). In detail, two flasks with 400 ml of Middlebrook 7H9 liquid medium supplemented with ADC enrichment were started with 4 ml of freshly prepared inoculum. Bacteria were grown at 37°C with aeration at 100 rpm. After four to five incubation weeks, the cell density was around 1 × 10^9^ CFU/ml (average, 800 mg of dry biomass per flask). The test culture (400 ml) was supplemented with usnic acid to a concentration of 512 μg/ml (normalized by weight, dissolved in DMSO), while the DMSO control (another 400-ml culture) was grown with 2% DMSO. The cultures were incubated for 24 h. The bacterial metabolism was stopped, and metabolites were quenched by addition of cold methanol (–60°C) (1:1, vol/vol). Next, the cultures were aliquoted in 50-ml Falcon tubes and centrifuged for 30 min at 8,000 rpm in 4°C. The supernatant was removed, and bacterial pellets were rinsed three times with cold phosphate-buffered saline buffer (Biomed, Lublin, Poland) and centrifuged again to remove traces of medium. The bacterial biomass was lyophilized, weighed, and stored at −60°C before analysis.

### Total RNA extraction.

M. tuberculosis cells were collected by centrifugation of 2 ml of a culture exposed to usnic acid. The same number of cells was harvested from DMSO-incubated culture. The total RNA extraction procedure followed Sawicki et al. (49). The bacterial pellet was resuspended with 1 ml of RNA pro solution (MP Biomedicals, Santa Ana, CA) and placed in a tube containing 0.8 ml zirconia beads (0.1-mm diameter). Cells were disrupted in a bead-beater (FastPrep24 instrument; MP Biomedicals, Santa Ana, CA) at the highest speed by two 45-s pulses. Cell remains were spun down, and the liquid was transferred to a new tube. Total RNA isolation was performed with the FastRNA Pro Blue kit (MP Biomedicals, Santa Ana, CA) according to the manufacturer’s manual. DNase I (MilliporeSigma, St. Louis, MO) was used to eliminate possible genomic DNA contamination of RNA preparations. RNA concentration and purity were determined by spectrophotometric measurement, and samples were aliquoted and stored at −80°C for future use.

### qPCRs.

All quantitative PCR (qPCR) reactions were performed according to Sawicki et al. (49) with a LightCycler EvoScript RNA SYBR green I master kit (Roche, Basel, Switzerland) in a LightCycler 480 thermal cycler (Roche, Basel, Switzerland) in a total volume per reaction of 20 μl on 96-well plates. The composition of the single reaction was 4 μl 5× master mix, 1 μl 20× primer mix (10 μM), 5 μl RNA template, and 10 μl PCR-grade water. Cycling conditions were reverse transcription at 60°C for 15 min, initial denaturation at 95°C for 10 min, 40 cycles of amplification at 95°C for 10 s, a 30-s incubation at 58°C, and melting curve from 25°C to 95°C with ramping rate of 2.2°C/s. For relative quantification of transcript levels, target genes were normalized to 16S rRNA. Primers were synthesized by MilliporeSigma and are listed in Table 3. Relative quantification was calculated according to the Pfaffl mathematical model (51).

**TABLE 3:** Primers used for qPCR analyses

Gene	Primer pair (5′–3′)
*sigA*	Fwd, GACGAAGACCACGAAGAC
	Rev, TCATCCCAGACGAAATCAC
*sigB*	Fwd, CTCGTGCGCGTCTATCTGAA
	Rev, AGCAGATGCTCGGCATACAA
*sigG*	Fwd, CGTCAATGAGCCTACGCAGA
	Rev, GCGAAATTCCGTTCAGTCCG
16S RNA	Fwd, ACTTCGGGATAAGCCTGGGA
	Rev, AGCGCTTTCCACCACAAGAC

### Metabolite extraction.

Chloroform-methanol (1:1; vol/vol) and methanol-water (1:1; vol/vol) were used for metabolite extraction from lyophilized bacterial biomasses for LC-MS and NMR analysis, respectively. Ten replicates of usnic acid-treated bacteria and 10 replicates of control samples were used for LC-MS analysis, while 5 replicates of usnic acid-treated bacteria and 5 replicates of control samples were used for NMR analysis. Each sample of dry biomass was poured with a mixture of solvents (1,500 μl) and sonicated for 20 min. After centrifugation (15 min at 13,000 rpm at 4°C), clear supernatants were transferred to clean Eppendorf tubes and evaporated under reduced pressure at 30°C to obtain dry extracts.

### HPLC-ESI-QTOF-MS analysis.

Metabolites extracted with a mixture of chloroform and methanol were dissolved in methanol-acetonitrile-isopropanol (1:1:0.5, vol/vol/vol) and separated on Gemini chromatographic column (3-μm-inner-diameter C_18_ with TMS endcapping, 110 Å, 150 by 2 mm) and guard column (Phenomenex Inc., Torrance, CA, USA). The separation was performed on an Agilent 1200 Infinity high-performance liquid chromatography (HPLC) chromatograph (Agilent Technologies, Santa Clara, CA). Metabolites detection and MS data acquisition were done on an Agilent 6530B Accurate-Mass quantitative time of flight (QTOF) spectrometer equipped with a dual Agilent jet stream spray source (ESI) (Agilent Technologies, Santa Clara, CA) connected to an N_2_ generator (generating N_2_ at purities of >99%; Parker Hannifin Corporation, Cleveland, OH) operating in the positive ion mode. LC and MS conditions were the same as published elsewhere (48).

### Computational analysis.

The acquired raw data were converted to mzDATA files using Mass Hunter qualitative analysis (version B.07.00; Agilent Technologies, Santa Clara, USA) and then processed for feature detection and discovery with the open-source software XCMS (version 3.7.1; https://xcmsonline.scripps.edu), applying the centWave algorithm. Normalization, scaling, and filtering were performed before statistical analysis. Intensity of 500 was set as a threshold for mass traces, which were retained only if present in at least 6 replicates. The correction of retention time was done by the obiwarp method. Feature annotation was performed with an allowed error of 5 ppm and absolute allowed error of 0.015 *m/z*. The database search was done with mass accuracy tolerance of 10 ppm. Features were found significant above a fold change of 1.5 and *P* value of 0.01. The metabolic model of M. tuberculosis H37Ra, MTUB419947, and BioCyc 19.5 was used for activity network analysis (mummichog version 1.1.6) with 5-ppm pathway deviation.

MS-LAMP software (http://ms-lamp.igib.res.in) with the integrated “M. tb Lipidome MS-LAMP” database was used for mycobacterial lipid characterization. The *m/z* values of detected features were assigned to singly protonated ions (M+H)^+^ with an allowed 0.05 *m/z* mass difference.

### NMR analysis.

Extracts obtained with a mixture of methanol and water were redissolved in 0.4 ml CD_3_OD and D_2_O, containing KH_2_PO_4_ buffer, pH 6.0, and TSPA-d4 as an internal standard at a final concentration of 0.005% (wt/vol). The samples were thoroughly mixed for 1 min and then placed in an ultrasonic bath (35 kHz) for 20 min at room temperature. After centrifugation (20 min, 12,000 rpm, 4°C), the supernatant was transferred to a 5-mm NMR tube (52). The NMR analysis was performed by following the protocol described by Georgiev et al. (52). The obtained 1D and 2D spectra were automatically reduced to ASC II files (AMIX software, v.3.7; Bruker, Karlsruhe, Germany) and further phase and baseline corrected and referenced at 0.0 ppm to the internal standard trimethylsilylpropanoic acid (TSPA) (MestReNova software, v. 12.0.0; Mestrelab Research, Santiago de Compostela, Spain). All signals in the spectrum were normalized according to the peak of TSPA and scaled to 1.0. The signals between δ 3.30 to 3.34 ppm and δ 4.80 to 4.88 ppm, belonging to the residual solvents, were excluded. The signals of usnic acid were also removed from the spectrum.

Further, OPLS-DA was performed (SIMCA-P software, v.15; Umetrics, Umeå, Sweden) using the scaling mode of Pareto (Par).

### Data availability.

Raw LC-MS and NMR data are deposited in figshare repository and are accessible at https://doi.org/10.6084/m9.figshare.14286326 and https://doi.org/10.6084/m9.figshare.14291772.

10.1128/mSystems.00097-21.1 Data Set S1 Fold change of individual lipids in classes/subclasses. Download Data Set S1, XLS file, 0.3 MB. Copyright © 2021 Sieniawska et al. 2021 Sieniawska et al. https://creativecommons.org/licenses/by/4.0/ This content is distributed under the terms of the Creative Commons Attribution 4.0 International license.

10.1128/mSystems.00097-21.2 Data Set S2 Pathway analysis results. Download Data Set S2, XLSX file, 0.01 MB. Copyright © 2021 Sieniawska et al. 2021 Sieniawska et al. https://creativecommons.org/licenses/by/4.0/ This content is distributed under the terms of the Creative Commons Attribution 4.0 International license.
